# Alternate-day dosing of pomalidomide in relapsed/ refractory multiple myeloma: a multicenter, single-arm phase 2 trial

**DOI:** 10.1038/s41375-023-01809-z

**Published:** 2023-01-12

**Authors:** Thilo Zander, Thomas Pabst, Sämi Schär, Stefan Aebi, Ulrich Mey, Urban Novak, Erika Lerch, Gaëlle Rhyner Agocs, Jeroen Goede, Zuzanna Maniecka, Stefanie Hayoz, Axel Rüfer, Christoph Renner, Christoph Driessen

**Affiliations:** 1 Division of Medical Oncology, Tumorzentrum, Luzerner Kantonsspital, Luzern, Switzerland; 2 Department of Medical Oncology, Inselspital, University of Bern, Bern, Switzerland; 3 Swiss Group for Clinical Cancer Research (SAKK) Coordinating Center, Bern, Switzerland; 4 Oncology and Hematology, Kantonsspital Graubünden, Chur, Switzerland; 5 EOC—Istituto Oncologico della Svizzera Italiana, Ospedale San Giovanni, Bellinzona, Switzerland; 6 Department of Medical Oncology, HFR Fribourg - Hôpital cantonal, Fribourg, Switzerland; 7 Kantonsspital Winterthur, Winterthur, Switzerland; 8 Division of Hematology, Tumorzentrum, Luzerner Kantonsspital, Luzern, Switzerland; 9 Onkozentrum Hirslanden Zürich, Zürich, Switzerland; 10 Department of Medical Oncology and Hematology, Kantonsspital St. Gallen, St. Gallen, Switzerland

**Keywords:** Myeloma, Drug development

## To the Editor:

Pomalidomide is a third-generation, oral immunomodulatory drug with activity in patients with relapsed/refractory multiple myeloma (rrMM). Pomalidomide (4 mg daily on a 21/28 day cycle), together with weekly dexamethasone, is an established standard treatment for rrMM, resulting in a remarkably consistent progression-free survival (PFS) of 4.0–4.6 months in two phase III trials, MM-003 and MM-010 [1, 2]. However, toxicity was considerable, with treatment-related grade 3/4 events in 60% of patients. Neutropenia (48% vs 16%) and pneumonia (13% vs 8%) were significantly more common with pomalidomide and low-dose dexamethasone than with high-dose dexamethasone alone in MM-003, leading to frequent treatment interruptions (66%) and dose reductions (24%) in a pooled analysis [3].

More recently, the addition of proteasome inhibitors or monoclonal antibodies to this pomalidomide-dexamethasone backbone has yielded superior myeloma control at the expense of increased toxicity [4, 5]. For example, a triplet combination incorporating the CD38 antibody isatuximab resulted in an 85% incidence of grade 3/4 neutropenia [5]. Such triplets currently represent the treatment standard for rrMM patients [6].

Thus, strategies to deliver pomalidomide + dexamethasone on a less toxic schedule are of interest. Lacy et al. tested two different pomalidomide total doses in a sequential non-randomized trial (4 mg for a 28/28-day cycle and 2 mg for a 28/28-day cycle) and observed similar activity of both dosing levels in dual-refractory myeloma patients, while the 2 mg dose appeared to have better tolerability [7].

Pomalidomide is commercially available as non-divisible capsules of 1, 2, 3, or 4 mg strength, respectively. The retail price of pomalidomide (950 USD per capsule, independent from capsule strength) links pomalidomide drug costs rather to the number of treatment days than to the actual drug dose delivered. As such, alternate-day treatment schedules may help to reduce the drug costs of pomalidomide treatment. Indeed, several aspects support that alternate-day pomalidomide dosing may be equally effective as standard daily dosing:

Pomalidomide has a longer initial plasma half-life (t_½_), compared to lenalidomide (7 vs 3 h), with a slow terminal-phase decline, particularly in MM patients [8]. A population pharmacokinetics analysis from the clinical trials CC-4047-MM-005/7 demonstrated a lack of correlation between the area under the curve of pomalidomide plasma concentration at steady state (AUC SS) and clinical efficacy endpoints (PFS and ORR) [9], consistent with the data from Lacy [7]. Because significant hematotoxicity of pomalidomide was already observed during early clinical development, Streetly et al. tested alternate-day administration in a dose-finding phase 1 study, demonstrating that it was associated with less myelosuppression while anti-myeloma activity was maintained with MTD for alternate day pomalidomide-dosing defined as 5 mg (28/28) [10].

Based on this, we tested the activity and safety of alternate-day dosing of 4 mg pomalidomide on a 28/28 day schedule in a multicenter, open-label phase 2 trial (OptiPOM; SAKK 39/16) in combination with low-dose weekly dexamethasone, in rrMM patients exposed to or intolerant to lenalidomide and bortezomib [11]. Eligibility criteria mirrored the earlier MM-003 trial: patients with measurable disease refractory to their most recent line of treatment (International Myeloma Working Group [IMWG] criteria) were included [12]. Participants had received ≥2 previous consecutive cycles of bortezomib and lenalidomide, alone or in combination, and prior treatment with an alkylating agent. The trial was conducted according to applicable regulations and approved by relevant institutional review boards or ethics committees and regulatory authorities, and all patients provided written informed consent (ClinicalTrials.gov Identifier: NCT03520985).

Treatment consisted of continuous oral pomalidomide 4 mg on alternate days of 28-day cycles, plus weekly oral dexamethasone 40 mg (or 20 mg if aged >75 years), until confirmed disease progression (PD) or intolerance. Prophylactic valaciclovir, cotrimoxazole, and acetylsalicylic acid were mandatory, while primary prophylactic granulocyte colony-stimulating factor (G-CSF) was not allowed (Supplementary Fig. 1). Efficacy assessments according to IMWG criteria were performed every 4 weeks during study treatment, safety assessments every 2 weeks.

The primary study endpoint was overall response rate (ORR), defined as minimal response or better (current IMWG criteria), confirmed by a central committee [13]. All authors had access to primary clinical trial data. Secondary endpoints were overall survival (OS), OS at 12 months, progression-free survival (PFS), and adverse events (AEs) recorded and graded according to the National Cancer Institute Common Terminology Criteria for Adverse Events (CTCAE v. 5.0.). Full source data verification was performed for the primary and secondary endpoints.

The trial was constructed by the Swiss Group for Clinical Cancer Research (SAKK) using a non-inferiority design, comparing the ORR of our alternate-day pomalidomide schedule versus standard-dose pomalidomide in MM-003 [1]. With a non-inferiority margin of 15%, and an alpha of 5%, 71 participants were required. Unfortunately, the study was prematurely terminated by the sponsor after the enrollment of 34 patients. The reasons for this were acute financial constraints at SAKK, together with slower-than-expected accrual because of increased availability of pomalidomide-containing triplets and the entire lack of external financial funding from commercial sources supporting this trial aiming to reduce pomalidomide drug treatment costs.

Due to premature trial termination, all analyses are descriptive without hypothesis testing. ORR was evaluated in all patients with valid response assessments. PFS (time from registration to disease progression/death) and OS (time from registration to death) were analyzed in an intent-to-treat Kaplan-Meier analysis. Observations were censored on December 31, 2020. Safety was analyzed in all patients who received ≥1 dose of study drug. Between October 2018 and October 2020, 34 patients (median age 75 years, range 52–87) were enrolled at 8 Swiss centers. Median time from myeloma diagnosis was 5.1 years (range 1.9–16.8 years). All patients had received lenalidomide, bortezomib, and an alkylating agent, with a median of 3 (range 2–8) prior lines of therapy. A significant proportion was pretreated with carfilzomib (29%) and daratumumab (27%). Fourteen (41%) patients had high-risk cytogenetic abnormalities (supplemental Table 1) [14].

Median treatment duration was 3.6 months, which was overall well tolerated. Toxicity consisted primarily of myelosuppression and infections (Table 1). Grade 3/4 neutropenia (24%) improved on pomalidomide dose delay/reduction, with therapeutic G-CSF being used in only 3 (9%) patients. We observed no neutropenic fevers or thromboembolic events. Renal function declined in 3 patients (9%; grade 3/4 in 2 patients [6%]). There were two on-study deaths: fulminant disease progression and sudden death (not considered treatment-related, in a patient with known cardiac disease).

**Table 1:** Treatment-related adverse events ≥3 during treatment phase in present OptiPOM study (alternate-day pomalidomide 4 mg; *n* = 34); to place results in context, treatment-related adverse events of participants in MM-003^a^ (standard pomalidomide 4 mg 21/28-day cycle arm; *n* = 302) have been added.

AE ≥ Grade 3	n (%) OptiPOM	n (%) MM-003^a^
Neutropenia	8 (24)	143 (48)
Anemia	6 (18)	99 (33)
Thrombocytopenia	4 (12)	67 (22)
Leukopenia	1 (3)	26 (9)
Infections	6 (18)	102 (34)
Pneumonia/Lung infection	3 (9)	42 (14)
Upper respiratory tract infection	1 (3)	5 (2)
Febrile neutropenia	0 (0)	28 (10)
Hypercalcemia	1 (3)	13 (4)
Hyperbilirubinemia	1 (3)	0 (0)
Fatigue/Malaise	2 (6)	16 (5)
Skeletal pain	3 (9)	36 (12)
Diarrhea	2 (6)	3 (1)
Skin rash	2 (6)	Not reported
Chronic kidney disease	2 (6)	Not reported

The ORR was 29.4% (95% confidence interval [CI] 16.9─44.8%), with 3 very good partial responses (VGPRs), 6 partial responses (PRs), and 1 MR; Fifteen patients (44%) achieved stable disease. Eight patients (24%) had PD after cycle 1 (Table 2). Median PFS was 4.2 months (95% CI, 1.9─5.5 months), and median OS not reached at the time of analysis (Supplemental Fig. 1). OS at 12 months was 66.5% (95% CI, 47.6─79.9). The median PFS of 4.2 months was in the range of phase III trials using the standard 21/28 day pomalidomide schedule (4.0─4.6 months) [1, 2]. Mateos et al. published a subgroup analysis of the OCEAN study of patients treated with the approved pomalidomide standard dosing: For this elderly patient group >65 years (n = 164), PR or better was 24%, median PFS 4.9 months. In our trial using the alternate-day schedule, patients had a median age of 75 years and PR or better was 26% [15].

**Table 2:** Responses and survival rates in present OptiPOM study (alternate-day pomalidomide 4 mg; *n* = 34); to place results in context, responses and survival rates of participants in MM-003^a^ (standard pomalidomide 4 mg 21/28-day cycle arm; *n* = 302) have been added.

	n (%) OptiPOM	*n* (%) MM-003^a^
Response to treatment		
Complete response (CR)	0 (0)	3 (1)
Very good partial response	3 (9)	14 (5)
Partial response	6 (18)	78 (26)
Minimal response	1 (3)	23 (8)
Stable disease (SD)	15 (44)	129 (43)
Overall response rate (MR or better; primary endpoint of the study)	10 (29)	118 (40)
Overall response rate (PR or better)	9 (27)	95 (32)
Progression-free survival; months		
Median	4.2	4.0
95% CI	1.9–5.5	3.6–4.7
Overall-survival at 12 months; %		
%	66.5	~55
95% CI	47.6–79.9	

Importantly, the rate of grade 3/4 events (infections, neutropenia, anemia, thrombocytopenia) of our alternate-day pomalidomide schedule appeared numerically almost 50% lower than that reported for trials using standard dosing, despite the lack of G-CSF prophylaxis and a higher proportion of patients aged >75 years. While mandatory antibacterial and antiviral prophylaxis may have contributed to the low number of infections, the low hematologic toxicity suggests that alternate-day pomalidomide dosing may be a valid strategy to improve safety.

Although our cohort differs from phase III trials in the same setting by a lower median number of prior lines of therapy (3 vs 5), the time from diagnosis was comparable. Conversely, the proportion of patients aged > 75 years or carrying high-risk cytogenetics was higher in OptiPOM, and a sizeable number of our patients had already been exposed to next-generation drugs such as carfilzomib and daratumumab.

The pharmacokinetics of pomalidomide is dominated by CYP1A2-mediated metabolism. The clinically most important and relevant inhibitors of CYP1A2 in this setting are fluoroquinolones (ciprofloxacin, ofloxacin), so their excess use could in theory explain the preserved efficacy of alternate-day pomalidomide dosing observed. However, antimicrobial prophylaxis was mandatory in our trial with cotrimoxazole, and neutropenic fever was not observed so it can be excluded that excess fluoroquinolone use may have biased the efficacy results in our cohort.

The interpretation of our trial data is limited by its premature closure which precludes comparison-based conclusions. However, the cohort of 34 patients treated with alternate-day pomalidomide dosing is the largest reported to date and is consistent with earlier trial data suggesting that pomalidomide treatment on alternate days is active and safe.

In summary, our OptiPOM data provide additional evidence that alternate-day pomalidomide dosing may be a reasonable, well-tolerated option to deliver pomalidomide, especially to patients at increased risk for treatment-related toxicity, while at the same time decreasing pomalidomide drug costs. The potential of alternate-day pomalidomide dosing schedules to improve the safety and cost-efficacy of pomalidomide-containing triplets should be clarified in a definite trial.

## Supplementary information

Supplemental Material_Final File

## Data Availability

Proposals for data access should be submitted to the corresponding author for consideration. Access to de-identified participant data can be granted if the proposal is approved by the Swiss Group for Clinical Cancer Research (SAKK).

## References

[1] Miguel JS, Weisel K, Moreau P, Lacy M, Song K, Delforge M (2013). Pomalidomide plus low-dose dexamethasone versus high-dose dexamethasone alone for patients with relapsed and refractory multiple myeloma (MM-003): a randomised, open-label, phase 3 trial. Lancet Oncol.

[2] Dimopoulos MA, Palumbo A, Corradini P, Cavo M, Delforge M, Di Raimondo F (2016). Safety and efficacy of pomalidomide plus low-dose dexamethasone in STRATUS (MM-010): a phase 3b study in refractory multiple myeloma. Blood.

[3] Moreau P, Dimopoulos MA, Richardson PG, Siegel DS, Cavo M, Corradini P (2017). Adverse event management in patients with relapsed and refractory multiple myeloma taking pomalidomide plus low-dose dexamethasone: a pooled analysis. Eur J Haematol.

[4] Richardson PG, Oriol A, Beksac M, Liberati AM, Galli M, Schjesvold F (2019). Pomalidomide, bortezomib, and dexamethasone for patients with relapsed or refractory multiple myeloma previously treated with lenalidomide (OPTIMISMM): a randomised, open-label, phase 3 trial. Lancet Oncol.

[5] Attal M, Richardson PG, Rajkumar SV, San-Miguel J, Beksac M, Spicka I (2019). Isatuximab plus pomalidomide and low-dose dexamethasone versus pomalidomide and low-dose dexamethasone in patients with relapsed and refractory multiple myeloma (ICARIA-MM): a randomised, multicentre, open-label, phase 3 study. Lancet (Lond, Engl).

[6] Dimopoulos MA, Moreau P, Terpos E, Mateos MV, Zweegman S, Cook G (2021). Multiple myeloma: EHA-ESMO Clinical Practice Guidelines for diagnosis, treatment and follow-up(†). Ann Oncol J Eur Soc Med Oncol.

[7] Lacy MQ, Allred JB, Gertz MA, Hayman SR, Short KD, Buadi F (2011). Pomalidomide plus low-dose dexamethasone in myeloma refractory to both bortezomib and lenalidomide: Comparison of 2 dosing strategies in dual-refractory disease. Blood.

[8] Li Y, Xu Y, Liu L, Wang X, Palmisano M, Zhou S (2015). Population pharmacokinetics of pomalidomide. J Clin Pharm.

[9] Li Y, Kassir N, Wang X, Palmisano M, Zhou S (2020). Population pharmacokinetics and exposure response analysis of pomalidomide in subjects with relapsed or refractory multiple myeloma from the novel combination treatment of pomalidomide, bortezomib, and low-dose dexamethasone. J Clin Pharm.

[10] Streetly MJ, Gyertson K, Daniel Y, Zeldis JB, Kazmi M, Schey SA (2008). Alternate day pomalidomide retains anti-myeloma effect with reduced adverse events and evidence of in vivo immunomodulation. Br J Haematol.

[11] Zander T, Aebi S, Pabst T, Renner C, Driessen C (2017). Spotlight on pomalidomide: could less be more?. Leukemia.

[12] Rajkumar SV, Harousseau J-L, Durie B, Anderson KC, Dimopoulos M, Kyle R (2011). Consensus recommendations for the uniform reporting of clinical trials: report of the International Myeloma Workshop Consensus Panel 1. Blood.

[13] Kumar S, Paiva B, Anderson KC, Durie B, Landgren O, Moreau P (2016). International Myeloma Working Group consensus criteria for response and minimal residual disease assessment in multiple myeloma. Lancet Oncol.

[14] Sonneveld P, Avet-Loiseau H, Lonial S, Usmani S, Siegel D, Anderson KC (2016). Treatment of multiple myeloma with high-risk cytogenetics: a consensus of the International Myeloma Working Group. Blood.

[15] Mateos M-V, Robak PJ, Rosiñol L, Symeonidis A, Sonneveld P, Thuresson M (2021). OCEAN (OP-103): melflufen/dexamethasone compared with pomalidomide/dexamethasone in patients with relapsed/refractory multiple myeloma-age subgroup analysis of older patients. Blood.

