# Supplementary material for: Alternate-day dosing of pomalidomide in relapsed/ refractory multiple myeloma: a multicenter, single-arm phase 2 trial
Source: Leukemia. 2023 Jan 12;37(3):699–701. doi: 10.1038/s41375-023-01809-z (PMC9991905; doi:10.1038/s41375-023-01809-z)

# Alternate-day dosing of pomalidomide in relapsed/ refractory multiple myeloma: a multicenter, single-arm phase 2 trial

# Supplement

## Supplementary Tables

## **Supplementary Table 1**

Patient Characteristics and previous therapies (n = 34); to place results in context, characteristics of participants in MM-003 pomalidomide + low-dose dexamethasone arm (n = 302) have been added

| **Characteristics** | **n (%) OptiPOM** | **n (%) MM-003** |
| --- | --- | --- |
| **Age, y** |  |  |
| Median | 75.0 | 64.0 |
| Range | 52-87 | 35-84 |
| **Sex** |  |  |
| Female | 17 (50) | 181 (60) |
| Male | 17 (50) | 121 (40) |
| **Time from diagnosis, y** |  |  |
| Median | 5.1 | 5.3 |
| Range | 1.9-16.8 | 0.6-30 |
| **N of prior lines of therapy** |  |  |
| Median | 3 | 5 |
| Range | 2 – 8 | 2 – 14 |
| **ECOG performance status** |  |  |
| 0-1 | 33 (97) | 248 (82) |
| >2 | 1 (3) | 52 (17) |
| **Creatinine clearance** |  |  |
| <60mL/min | 14 (41) | 95 (31) |
| **Simplified frailty score** |  |  |
| Frail | 25 (74) | unknown |
| non-frail | 9 (26) | unknown |
| **High-risk cytogenetic abnormalities** |  |  |
| Yes | 14 (41) | unknown |
| No | 9 (26) | unknown |
| Unknown | 11 (32) |  |
| **Previous treatments** |  |  |
| Lenalidomide | 34 (100) | 302 (100) |
| Bortezomib | 34 (100) | 302 (100) |
| Carfilzomib | 10 (29) | n/a |
| Daratumumab | 9 (27) | n/a |
| **Refractory to** |  |  |
| Lenalidomide | 19 (56) | 286 (95) |
| Bortezomib | 14 (41) | 238 (79) |
| Bortezomib and lenalidomide | 13 (38) | 225 (75) |

n/a, not applicable

## Supplementary Figures

## Supplemental Figure 1.

**Phase 2 trial of alternate-day pomalidomide and low-dose dexamethasone in relapsed/refractory MM.**

(A) Treatment schema including prophylaxis (B) Progression-free survival and (C) Overall survival, by Kaplan-Meier analysis.


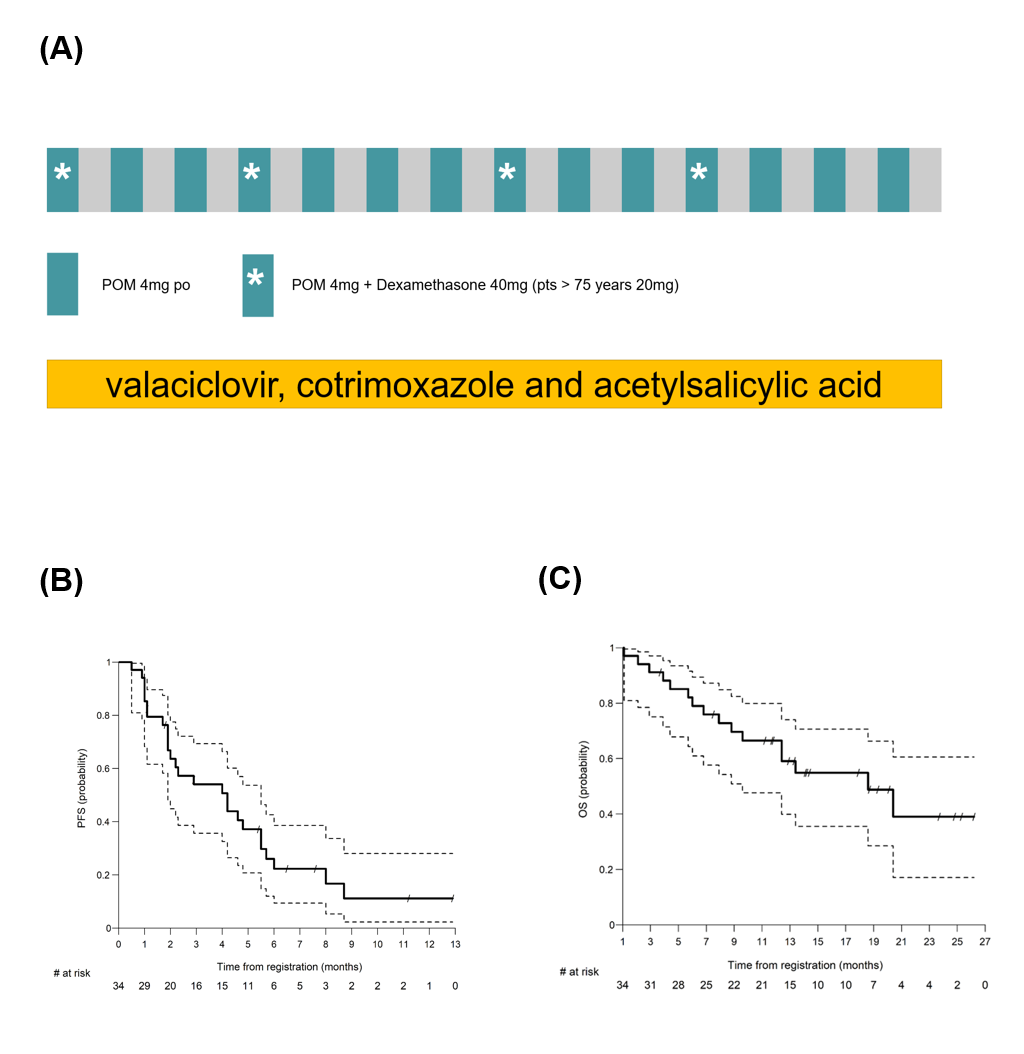

Supplement: Supplementary file 1 — Supplemental Material_Final File [file 41375_2023_1809_MOESM1_ESM.docx]
